# Contextual influences on the choice of long-acting reversible and permanent contraception in Ethiopia: A multilevel analysis

**DOI:** 10.1371/journal.pone.0209602

**Published:** 2019-01-16

**Authors:** Yohannes Dibaba Wado, Eshetu Gurmu, Tizta Tilahun, Martin Bangha

**Affiliations:** 1 African Population and Health Research Center, Manga Close, Kitisuru, Nairobi, Kenya; 2 Center for Population Studies and Institute of Development and Policy Research, Addis Ababa, University, Addis Ababa, Ethiopia; University of South Carolina Arnold School of Public Health, UNITED STATES

## Abstract

**Background:**

Long acting reversible and permanent contraception (LARPs) offer promising opportunities for addressing the high and growing unmet need for modern contraception and helps to reduce unintended pregnancies and abortion rates in sub-Saharan Africa (SSA). This study examines the contextual factors that influence the use of long acting reversible and permanent contraception among married and fecund women in Ethiopia.

**Method:**

We use data from the 2016 Ethiopian Demographic and Health Survey to examine the contextual factors that influence choice of long acting reversible and permanent contraception among married, non-pregnant and fecund women. The DHS collects detailed information on individual and household characteristics, contraception, and related reproductive behaviors from women of reproductive age. In addition, we created cluster level variables by aggregating individual level data to the cluster level. Analysis was done using a two-level multilevel logistic regression with data from 6994 married **(**weighted = 7352) women residing in 642 clusters (communities).

**Results:**

In 2016, 12% of married, non-pregnant and ‘fecund’ women were using long-acting reversible and permanent methods of contraception in Ethiopia. A higher proportion of women with secondary and above education (17.6%), urban residents (19.7%), in the richest wealth quintile (18.3%) and in paid employment (18.3%) were using LARP methods compared to their counterparts. Regression analysis showed that community level variables such as women’s empowerment, access to family planning information and services, region of residence and knowledge of methods were significantly associated with use of LARP methods. Age, wealth status, employment status and women’s fertility preferences were among the individual and household level variables associated with choice of LARP methods. With regards to age, the odds of using LARP methods was significantly lower among adolescents (OR, 0.53; 95% CI, 0.32–0.85) and women over the age of 40 (OR, 0.63; 95% CI, 0.44–0.90) compared to women in their 20’s.

**Conclusion:**

The findings of this study indicate that the demand for long-acting reversible and permanent contraception is influenced not only by women’s individual and household characteristics but also by the community’s level of women’s empowerment, socio-economic development, as well as access and exposure to family planning information and services. Thus, improving knowledge of long-acting reversible and permanent methods, improving women’s decision making autonomy and upgrading the capacity and skills of health workers particularly the midlevel providers and community health extension workers on the provision of LARP methods and rights-based approach is important to improve the uptake of LARP methods.

## Introduction

Contraceptive prevalence increased worldwide from about 36% in 1970 to 63% in 2017 [1], yet a staggering 214 million women in low and middle income countries (LMICs have unmet need for modern contraception [2]. In Sub-Saharan Africa (SSA), nearly 1 in 4 married women have unmet need for contraception. As a result of the high unmet need for contraception, it is estimated that more than 80 million unintended pregnancies and about 25 million unsafe abortions occur annually in the world [3–6]. For instance, recent analysis show that abortion rates and annual number of induced abortions in SSA increased between1990 and 2014 [4, 6, 7]. While the reasons for unmet need are diverse ranging from fear of side effects of using contraception to lack of access to contraception and opposition to use [7], long acting reversible and permanent (LARPs) contraception offer promising options for addressing the high unmet need for modern contraception in sub-Saharan Africa.

LARP methods (including IUD, implants and permanent sterilizations) are highly effective, have minimal adherence requirements, and offer high rates of patient satisfaction and continuation [3, 8, 9]. As such, increased use of LARP methods has the potential to reduce unintended pregnancies and abortion rates. Moreover, by improving method choice, LARP methods offer opportunities for rights based family planning programming. A rights based family planning is ensuring that people have the information and access to a full range of safe and effective contraceptive methods to exercise free and informed reproductive choices [10, 11]. LARPs are also cost effective in terms of the cost per Couple Years of Protection (CYPs). Thus promoting LARPs at this critical time of dwindling funding for family planning offer greater opportunities to domesticate budgets for family planning.

Evidences show that broader contraceptive method mix expands contraceptive choice and improves family planning use [8, 12, 13]. Studies of contraceptive method choice in developing countries showed that contraceptive prevalence is highest in countries where access to all methods is uniformly high [13, 14]. In a study of Implants in Africa, Jacobstein (2018) found that increased implant use has been the main driver of the increased modern contraceptive prevalence attained by 11 countries in the region, with gains in Implant use alone exceeding combined gains in use of injectables, pills, and IUDs [8]. Jane and colleagues (2013) also showed that full availability of one additional method was associated with an eight percentage point reduction in the level of contraceptive discontinuation that in turn increase unmet need [15].

Method specific prevalence of contraception varies across regions, with female sterilization and IUDs being the most commonly used methods in Asia, Latin America, North America and Europe [3, 16]. However, LARP methods are the least common in SSA where the use of injectable and traditional methods are more common. Little is known about the reasons for the low uptake of long-acting reversible and permanent contraception in SSA. But, studies attribute the relatively low uptake of long-acting reversible methods to clients’ lack of knowledge, dependence on the provider for information, previous negative experiences and provider bias for permanent contraception among others [17–20]. Women’s misconceptions of contraceptive side–effects, partner disapproval and socio-cultural norms of fertility also influence choice of LARP contraception [21, 22].

There have been few studies that examined how contraceptive behaviors are influenced by factors beyond the individual and household-level [23, 24]. The few studies that examined the effects of contextual factors [23, 25] suggest that availability of reproductive health services, quality of services, cultural beliefs, and women’s empowerment influence use of modern contraception. Stephenson and colleagues examined the role of community-level factors in explaining geographic variations in modern contraceptive use in six African countries and found that the range of these factors associated with contraceptive use varied across the six countries[23]. Analysis of Demographic and Health Surveys (DHS) from Rwanda and Nepal found that the community’s level of socioeconomic development, the extent to which women in the community participate in decision-making around family planning, prevailing small-family size norms, and the community’s access to modern methods were positively associated with women’s contraceptive use [25].

However, very few of these studies specifically focused on the effects of contextual factors on the choice of LARP methods. Ethiopia, with a rapidly rising contraceptive prevalence and programmatic focus on expanding access to LARP methods provides a unique context for studying the role of contextual community level factors in the uptake of LARP methods. This study investigates the extent to which contextual individual, household and community level factors of women’s empowerment, access to family planning information and services, community level fertility norms and socio-economic development shape women’s choice of LARP methods in Ethiopia.

### Ethiopia context

Ethiopia, the second populous country in Africa with an estimated population of about 105 million in 2107 [26], has recently experienced progressive declines in fertility. The total fertility rate (TFR) has declined to 4.6 children per women in 2016 from 5.5 in 2000 [27, 28]. Historically Ethiopia had one of the lowest contraceptive prevalence rate with little or no consideration to issues of contraceptive method mix. Family planning services began early in 1966 with the establishment of the Family Guidance Association of Ethiopia (FGAE), an International Planned Parenthood Federation affiliate [29]. In the 1980s, the Ministry of Health (MOH) added family planning to its maternal and child health program but family planning and population issues remained unpopular until the promulgation of a national population policy in 1993. The objectives of the population policy included increasing the prevalence of contraceptive use (CPR) to 44% by 2015 [30],

Ethiopia experienced remarkable progress in improving access to family planning since 2000 although contraceptive method mix has been dominated by one method, injectable. The use of modern contraception increased from 6.3% in 2000 to 35% in 2016 [27, 28]. Analysts have attributed the success of Ethiopia’s family planning programs to four important and interrelated factors: Political will, donor support, non-governmental and public-private partnerships and the government’s establishment of a network of Health Extension Workers [29, 31]. Olson and Piller (2013) provided a detailed review of the contribution of each of these four factors to the growth of family planning in Ethiopia, of which we echo the role of the Health Extension Program (HEP).

The HEP was launched in 2003 as part of the second five year Health Sector Development Program (HSDP) to increase access to promotive and preventive health services including family planning methods to the largely rural population of Ethiopia. Every village (Kebele) has a health post staffed with at least two female health extension workers (HEWs) who spend most of their working days in the community conducting house to house visits to teach and counsel women and family members on health promotion and prevention of diseases [32]. They help to generate demand for family planning, address misconceptions about family planning, counsel clients on the family planning methods, provide methods available at the health posts and refer clients to health centers in cases of extended needs. They provided mainly short term family planning methods (pills, condoms and injectables) but since 2009, with additional training, HEWs were able to insert implanon at the community health post under close supervision of health workers in the nearest health centers.

Building on the Population Policy and National Health Policy, the Ministry of Health developed a Reproductive Health Strategy (2006–2015) in 2006 which was updated in 2016 (2016–2020). While these previous policies and strategies gave little attention to the promotion of long acting reversible and permanent contraception, the revised reproductive health strategy of 2016 clearly articulates the government’s commitment to expanding access to LARP methods with as target to account for half of method mix by 2020 [33]. The Ministry also developed and costed the implementation plan for achieving the targets set in the strategy[34]. Among the efforts being made to attain this target includes an ongoing task shifting and task sharing with the implanon scale up program, IUD revitalization program in which HEWs and mid-level providers play a major role through demand creation, referral and insertion of implants and IUD [35].

Despite the rapid increase in contraceptive use, several challenges remain. There is a highly skewed method mix dominated by injectables, poor informed choice and higher contraceptive discontinuation rate [28]. Data on method information index shows that only 30% of users reported receiving adequate information for informed choice and nearly 35% of users discontinue a method within the first year of acceptance Above all, wider geographic and socio-economic disparities in contraceptive use exist [28]. Understanding the contextual factors that shape the uptake of LARP methods is thus important for programmatic interventions that aim at a rapid scale up of LARP methods, improve method choice and reduce contraceptive discontinuation rates.

## Methods

### Data source

This study uses data from Ethiopia Demographic and Health Surveys (DHS) conducted by the Ethiopian Central Statistical Agency (CSA) and ICF International. DHS is a nationally representative household survey of cross-sectional design. DHS surveys collect detailed information on individual and household characteristics, fertility, sexual behavior, contraception, and related reproductive behaviors from women of reproductive age (15–49 years). The latest 2016 DHS was used to examine the contextual factors that influence choice of long acting reversible and permanent contraception in Ethiopia.

In all the surveys, a two-stage stratified sampling is employed. At stage one, regions are stratified into urban and rural areas. Enumeration areas or clusters from each region proportional to the size of the national population were selected. In sampled households, all women aged between 15 and 49 years who consent to participate in the survey were interviewed. In the 2016 Ethiopian DHS, a total of 16,650 households and 15,683 women participated–with a reasonably high response rate of 98% and 95% respectively [28]. This study draws a sub-sample of married women of age 15–49 from the national sample and included a total of **6994 *married women* (weighted = 7352)** of age 15–49 who were not pregnant and not infecund at the time of the survey.

The DHS included several questions that differentiate the exposure status of women; whether pregnant, postpartum amenorrheic, menopausal, infecund or fecund. Women are considered fecund at the time of the survey if they have been married or in union, are not menopausal and not postpartum amenorrheic and not pregnant but have had birth in the five years preceding the survey [28]. Sexually active unmarried young women (15–24) constituted a very small proportion (less than 8% of young women 15–24 years) due to low reports of sexual activity and so were not included. Moreover, women who reported they were infecund and menopausal at the time of the survey were also excluded.

### Variables

The outcome variable in study is current use of long acting reversible and permanent contraceptive methods–a dichotomous variable coded ‘1’ if women or their partners were using implants, IUD and female or male sterilization and ‘0’ otherwise. Those coded as ‘otherwise’ include nonusers of contraception and users of short acting family planning methods (including injectables, pills, condom and natural methods). Several variables that have been associated with contraceptive use in literature were included as independent variables. We categorized them into three sets: individual and household levels, health facility and community-level variables.

Individual and household level variables in this analysis represent woman and household specific characteristics. These include age of women, education, religion, urban or rural residence, household wealth index, employment status, parity and fertility preferences. We recoded women’s age in to four categories (15–19, 20–29, 30–39 & 40–49 years). Education is recoded into three categories as; ‘no education’, ‘primary’ and ‘secondary and above’. The household wealth index is a composite variable based on data on household possessions and amenities collected in DHS surveys. This index categorizes women into five categories (poorest, poorer, middle, richer and richest). In the DHS, women are considered as employed if they had done any work other than their regular housework in the 12 months preceding the survey whether they paid or not. We then regroup women’s employment status into three categories: unemployed, paid employment, and unpaid employment. Parity is also recoded into four categories as the distribution of women by their number of living children (0/1, 2–3, 4–5 and 6^+^). Women’s fertility preference is recoded into four categories: desire for a child soon, desire more children later, desire no more children and undecided.

Access to family planning information was measured using the percentage of women in the cluster who heard about family planning messages through radio, television, newspapers or magazines or through mobile phones in the few months preceding the survey. Moreover, the DHS also asks women whether they were contacted by community health workers or family planning providers in the year before the survey. Access to family planning services was difficult to measure as the DHS did not include any direct question on access to services. In this paper, we used proxy variables of barriers to medical care—the DHS question on whether distance to facility is a big problem or not as a proxy for ‘distance to health facility’.

Other contextual community level variables considered include community level women’s empowerment, fertility norms, poverty. We created these cluster level variables by aggregating individual level data to the cluster level. These include *cluster level poverty*, which was created from the mean values of wealth index categories of the individual households aggregated at the cluster level, cluster level education created by aggregating values of the educational level of women in the cluster and a new category of regions, development region, based on their similarities in terms of economic activities, settings and socio-economic characteristics. *Community level women empowerment* variable was created by aggregating a composite variable constructed from variables on household and women’s health care seeking decisions. The composite variable indexes the proportion of women who participated in the decisions jointly with their husbands or alone. The variable *community level fertility norms* was constructed by aggregating the number of children desired at the cluster level. Similarly, the variable *community level marriage norm* was constructed by aggregating median age at marriage at the cluster level. The variable ‘development region’ is created by categorizing regions based on their dominant economic activity and based on the Ethiopian government’s classification of region into: ‘urban’ ‘agrarian’ and ‘emerging’ regions [35]. The first category (urban) includes the city administrations of capital Addis Ababa and Dire Dawa which are dominantly urban. The second category included dominantly rural regions with better distribution of health facilities and includes Amhara, Oromia, Tigray, and Southern Nations, Nationalities and People’s region and Harari. The remaining four regions (Afar, Somali, Benishangul Gumuz and Gambella) are not at the same level with the other regions in terms of provision of and access to healthcare and are thus classified as ‘emerging’ regions.

### Data analysis

Data were analyzed using STATA software version 14. First, we conducted exploratory analyses of each of the variables as well as descriptive analysis looking at trends and patterns in the use of modern contraception. Bivariate regression analysis was done to examine associations between contraceptive use and the selected individual, household and community level variables. Variables were included into the multivariate mixed effects logistic regression if they had a significant association at the bivariate level (P-value <0.05). Multilevel mixed effects logistic regression analysis was used to identify the net effects of each explanatory variable after adjusting for potential confounders.

Given the hierarchical nature of the DHS data, we used a multilevel logistic regression analysis to capture covariance at the individual/household and cluster level. Multilevel modelling is a robust method for analyzing hierarchically clustered data such as the DHS [36]. It provides a mechanism for measuring the influence of community factors and unobserved community effects on health outcomes. Moreover, multilevel modelling corrects the estimated standard errors to allow for clustering of observations within units. We reported fixed effects odds ratios (OR) and 95% confidence interval (CI) after controlling for the effects of individual and household characteristics. Statistical significance was determined using two tailed Wald test with the significance level of alpha of 5%. We used Hosmer and Lemshaw goodness of fit statistics to check the fitness of the full model (model IV) and the result showed that the model fit was good. The random effects were expressed in terms of community level variance (*δ*2). Intra-class correlation coefficient (ICC) was used to examine clustering and the extent to which contextual factors explain the unexplained variance of the empty model.

## Results

The socio-demographic characteristics of married women in the sample is shown in Table 1. The mean age of women in the study was 29.5 years with larger proportion of women falling in the age groups of 20–29 and 30–39 years. A smaller proportion, only 6.3% were adolescents. The majority of women (84%) resided in rural areas, had no formal education (61%), were not employed (52%), and had 2 or more living children. The mean number of living children to women in the sample was 2.4 with some 22% of women having 6 or more children (Table 1).

**Table 1:** Socio-demographic characteristics of married and fecund women, Ethiopia 2016.

characteristics	Frequency	Percentage
**Age**		
15–19	461	6.3
20–29	3186	43.3
30–39	2756	37.5
40–49	951	12.9
Mean (SD)		29.47 (SD±7.4)
**Educational status**		
No education	4485	61.0
Primary	2070	28.2
Secondary & higher	797	10.9
**Residence**		
Urban	1164	15.8
Rural	6188	84.2
**Wealth index**		
Poorest	1442	19.6
Poorer	1498	20.4
Middle	1511	20.6
Richer	1404	19.1
Richest	1497	20.4
**Employment status**		
Not employed	3824	52.0
Employed not for cash	2060	28.0
Employed for cash	1467	20.0
**Number of living children**		
0/1	1732	23.6
2–3	2185	29.7
4–5	1843	25.1
6+	1593	21.7
Mean (SD)		2.4(SD ±1.07)
**Total**	**7352**	**100**

**Fig 1:**
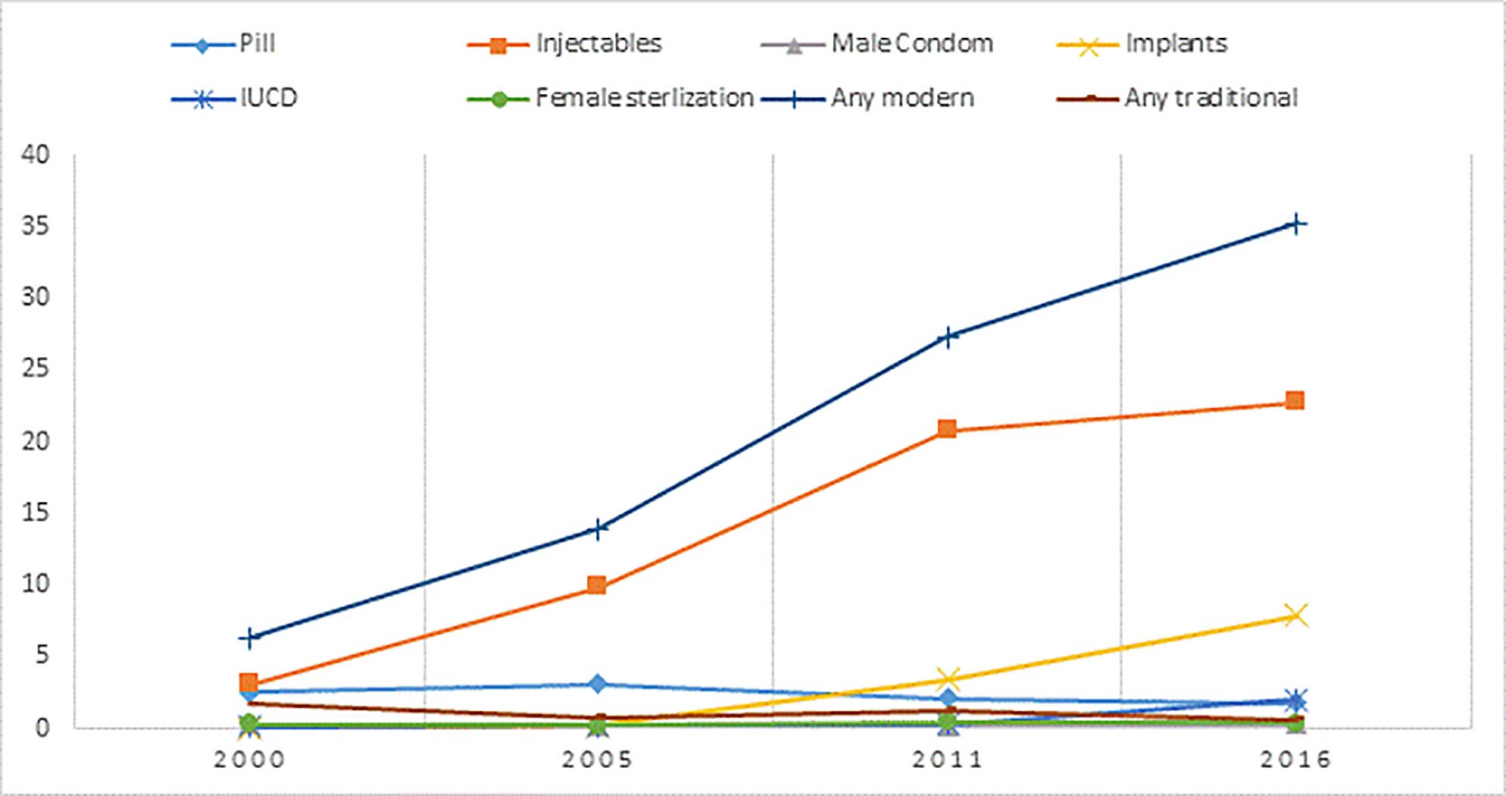
Trends in contraceptive method mix in Ethiopia: 2000–2016.

Fig 1 shows trends in contraceptive method mix in Ethiopia from 2000 to 2016. Modern contraceptive use has rapidly increased in Ethiopia in the last one and half decades, from a mere 6% in 2000 to 28% in 2011 and further to 35% in 2016 with an average increase of about 2% per annum. Of all methods, the one with the fastest increase is injectables whose share of contraception rose from 38% of method mix in 2000 to 73% in 2011 although it declined to 64% in 2016. During the same time, the share of LARP methods (implants, IUD, female sterilization and male sterilization) rose from a mere 4% in 2000 to 15% in 2011 and further to 29% in 2016. Among LARP methods, the use of implants has grown from almost none in 2000 to 22% of method mix in 2016 as the government’s family planning programs prioritized a rapid scale up of implants. The proportion of women who choose IUD has also increased recently although still contributing to only 2% of the method mix. In Ethiopia, the use of traditional methods has been low compared to many other countries in Africa (Fig 1).

However, disparities in the use of modern contraception and specifically uptake of long acting reversible and permanent contraception was evident by region, by place of residence, wealth quintile, women’s education, age, employment and other factors. (Table 2). Overall, 12.3% of married, non-pregnant and fecund women were using long acting reversible and permanent contraception at the time of the survey, while nearly 30% of women used short acting methods. A higher proportion of women with secondary and above education (17.6%), urban residents (19.7%), women from the richest households (18.3%) and women in paid employment (18.3%) used LARP methods compared to their counterparts (Table 2). Relatively higher proportion of women who heard family planning information on the media used LARP methods compared to those who have not heard such information in the last 12 months (Table 2).

**Table 2:** Distribution of currently married and fecund women by type of family planning use, Ethiopia 2016 (N = 6994).

characteristics	Not using	Using short acting methods	Using LARP methods	p-value
**Age**				0.001
15–19	66.9	26.9	6.2	
20–29	54.6	32.4	13.0	
30–39	60.1	26.6	13.3	
40–49	58.0	31.9	10.1	
**Educational status**				0.001
No education	63.0	25.1	11.9	
Primary	54.6	34.2	11.2	
Secondary & higher	37.6	44.9	17.6	
**Residence**				0.001
Urban	36.7	43.6	19.7	
Rural	61.9	27.2	10.9	
**Wealth index**				0.001
Poorest	76.9	16.9	6.2	
poorer	63.4	26.3	10.3	
Middle	57.0	30.0	13.0	
Richer	51.1	35.3	13.6	
Richest	41.3	40.4	18.3	
**Employment status**				0.001
Not employed	62.5	27.6	10.0	
Employed not for cash	56.2	31.5	12.4	
Employed for cash	48.4	33.4	18.3	
**Fertility preferences**				0.001
Wants a child soon	70.2	20.8	8.9	
Wants later	55.4	32.3	12.3	
Wants no more	54.0	32.6	13.8	
Undecided/unsure	62.9	29.8	12.3	
**Number of living children**				0.001
0/1	52.2	35.6	12.3	
2–3	52.2	32.7	15.2	
4–5	59.9	27.5	12.7	
6+	69.6	22.4	8.0	
**Heard about FP on media in the last few months**				0.001
**No**	61.9	27.2	10.9	
**Yes**	47.6	36.6	15.8	
**Visited by CHW**				0.001
**No**	60.5	27.8	11.8	
**Yes**	52.0	34.5	13.5	
Total	**57.9**	**29.8**	**12.3**	

**Fig 2:**
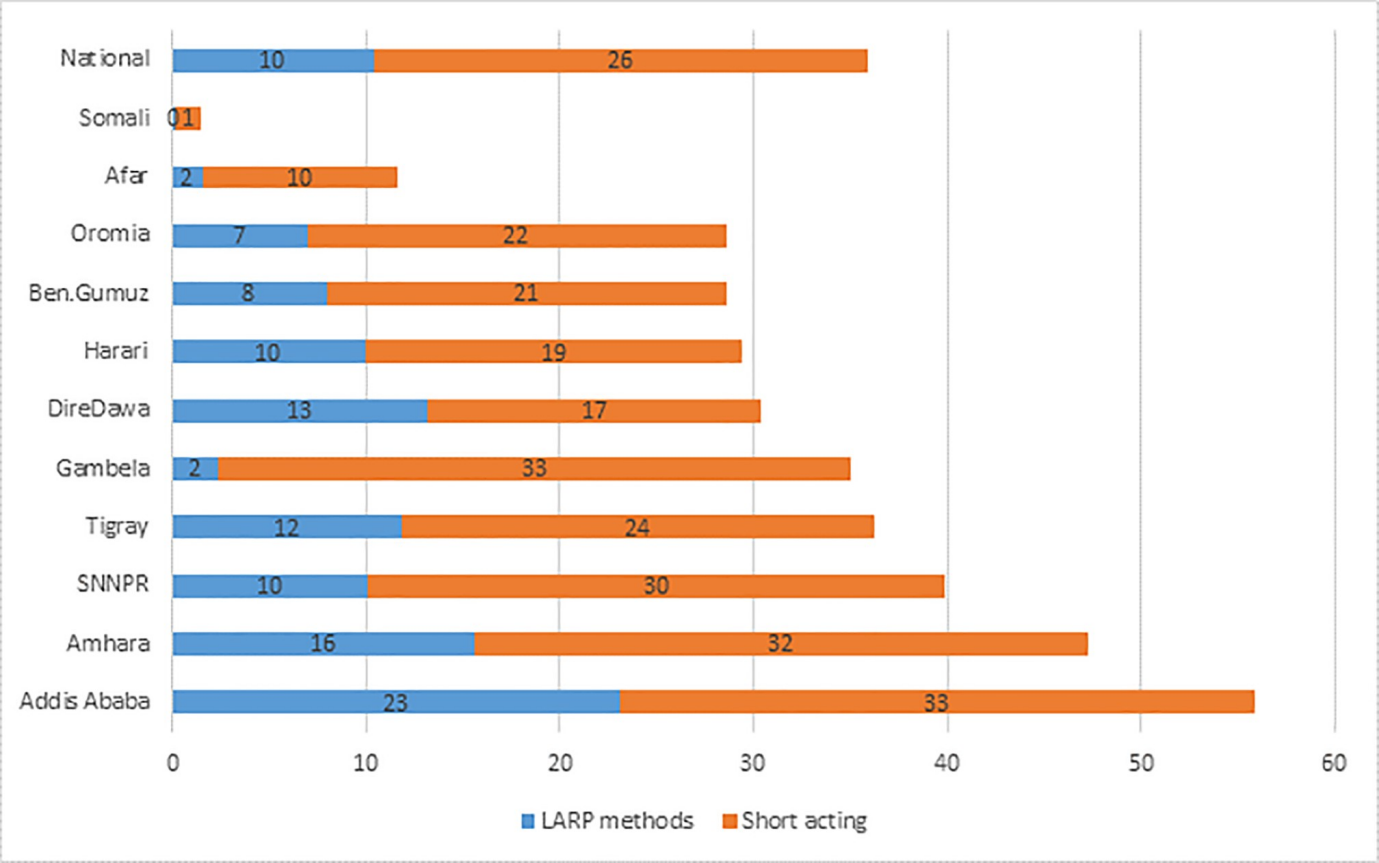
Regional variation in contraceptive use and method choice, Ethiopia 2016.

Variation in uptake of LARP methods is also observed with the number of living children, women’s fertility preferences and whether they were visited by community health workers or not (Table 2). The proportion of married women who used long acting reversible and permanent methods ranged from less than 1% in the Somali Region, a dominantly pastoral region to 23% in Addis Ababa (Fig 2).

### Multilevel regression analysis

Table 3 shows results of the multi-level logistic regression analysis of the association of LARP methods with several individual, household and community level contextual factors (Models I-IV). Bivariate regression analysis was conducted to test the association between the outcome and explanatory variables, and variables were included in to the multivariate multi-level regression analysis based on their association at the bivariate level (S1 Table). Models I-III present results of the different individual, household and community level variables while model IV shows results of all variables included in the multilevel regression model. The results show that there is a considerable level of variation in the uptake of LARP methods at the individual, household and community levels. As reported by intra-cluster coefficient (ICC) nearly 34% of the variation in the uptake of LARP methods is attributed to whether a woman resides in one community or another (Table 3, model I). The variation in use of LARP methods was reduced by the addition of contextual factors. Nonetheless, the community and health facility variables included in the analysis do not fully explain the community-level variation in contraceptive use as several health facility and quality of care related variables were not measured through the DHS.

**Table 3:** Multilevel analysis of the association between individual, household and cluster level variables with use of long acting and permanent contraception, Ethiopia 2016.

Variables	Model I	Model II	Model III	Model IV
**Individual and household variables**				
**Age** (ref = 20–29 years				
15–19		0.47(0.28–0.77)**		0.53(0.32–0.85)**
30–39		1.03(0.82–1.30)		0.92(0.73–1.16)
40–49		0.75(0.52–1.07)		0.63(0.44–0.90)**
**Education** (ref = no education)				
Primary education		0.90(0.71–1.13)		0.85(0.68–1.07)
Secondary and above		0.92(0.69–1.25)		0.87(0.66–1.18)
**Residence** (RC = urban)				
Rural		0.68 (0.45–1.00)		0.93(0.50–1.76)
**Wealth** (ref = poorest)				
poorer		2.30(1.62–3.30)*		1.52(1.06–2.18)*
middle		3.07(2.15–4.39)**		1.87(1.31–2.68)**
richer		2.96(2.04–4.31)**		1.77(1.21–2.59)**
richest		3.65(2.34–5.70)**		1.91(1.20–3.07)**
Participation in household decisions (ref = no)				
Participates in decisions		1.21(0.98–1.49)		1.08(0.87–1.34)
**Employment** (ref = not working)				
working but not paid in cash		1.24(0.98–1.56)		1.17 (0.93–1.47)
working and paid in cash		1.51(1.22–1.88)**		1.41(1.13–1.74)**
**Parity** (ref = = up to1 child)				
2–3		0.98 (0.77–1.25)		1.07(0.84–1.37)
4–5		0.81(0.56–1.12)		0.95(0.68–1.33)
6+		0.70(0.47–1.05)		0.91(0.60–1.37)
Fertility preference (ref = Want soon)				
Wants later		2.34(1.81–3.04)**		2.15(1.66–2.80)**
Wants no more		2.83(2.12–3.78)**		2.45(1.83–3.29)**
Undecided/unsure		3.01(1.95–4.66)**		2.70(1.73–4.20)**
**Access to family planning information and services**				
Exposure to family planning information (RC = no)				
Yes			1.24(1.01–1.53))*	1.24 (1.00–1.54))*
Visited by FP worker in the last 12 months (RC = no)				
Yes			1.04(0.86–1.25)	0.97(0.82–1.16)
Distance to health facility (Rc = not a big problem)				
Distance is a big problem			1.20(0.98–1.47)	1.21 (0.99–1.48)
Knowledge of LARP methods (Rc = low)				
High knowledge			4.95(3.34–7.33)**	4.19 (2.83–6.20)**
**Community**/***Cluster variables***				
Development region (RC = rural agrarian)				
Emerging/nomadic regions			0.39(0.27–0.56)**	0.45(0.31–0.64)**
Urban			1.09(0.77–1.88)	1.25(0.88–1.75)
Community poverty (RC = high)				
Low poverty			1.14 (0.76–1.71)	0.91(0.46–1.80)
Community level women’s education (RC = Low)				
High			1.06(0.75–1.51)	1.06 (0.74–1.51)
Women’s empowerment (Rc = low)				
High empowerment			1.48(1.11–1.97)**	1.37 (1.03–1.83)**
Community level fertility norms (Rc = high desired # of children)				
Lower desired # of children			0.81(0.62–1.05)	0.87(0.67–1.13)
Median at marriage (RC<17.0 years)				
Median age above 17 years			1.06(0.81–1.39)	1.05(0.80–1.38)
Variance	1.68	0.927	0.805	0.645
ICC	0.338	0.220	0.194	0.164
n	6994	6994	6994	6994
Number of clusters	642	642	642	642

*significant at P<0.05

**significant at P<0.01

The regression analysis identified several individual, household and community level factors that influence the uptake of LARP methods. Among individual and household factors, age of women, wealth status, women’s employment and fertility preference were significantly associated with use of LARP methods. The odds of using LARP methods was significantly lower among adolescent of age 15–19 (OR, 0.53; 95% CI, 0.32–0.85) and older women of age 40–49 (OR, 0.63; 95% CI, 0.44–0.90) compared to women in their 20’s. Women’s education and place of residence did not show significant association in all the models. The odds of using LARP contraception increased as household wealth status increased. For instance, the odds of using LARP methods was nearly twice (OR, 1.91; 95% CI, 1.20–3.06) higher among women in the richest wealth category compared to those in the poorest quintile. Likewise, the odds of using LARP methods was 41% higher (OR, 1.41; 95% CI, 1.14–1.75) among women employed with cash income compared to those who were not working after adjusting for the effects of individual, household and community level variables. Among individual factors, women’s fertility preference was a stronger predictor of the choice of LARP methods. The odds of choosing LARP methods was more than twice higher among women who want to space, who want to limit or women who were not sure of the timing of their next child compared to those who desire to have a child soon (Table 3).

The study focuses on the role of cluster level variables in explaining the between cluster variability in the use of LARP methods. Model III shows that among the cluster level variables: exposure to family planning information, knowledge of LARP methods, region and women’s empowerment were significantly associated with use of LARP methods (Model III). After adjusting for the effects of individual and household factors, few contextual community level factors appeared significantly associated with use of LARP methods (Model IV): exposure to family planning information on media, knowledge of LARP methods, region and women’s empowerment were significantly associated with choice of LARP methods. Exposure to family planning information through media had a significant positive effect, with higher odds (OR, 1.24; 95% CI, 1.00–1.54) of using LARP methods among women with exposure to family planning information on the media compared to those who did not. Perceived distance to health facility had a marginal positive effect (OR, 1.21;95%CI,0.99–1.48) on the use of LARP methods with women who reported distance being not a big problem having higher odds of choosing LARP methods compared to those who reported distance is a big problem.

Women empowerment at the community level has shown significant association with the choice of LARP methods after adjusting for the effects of individual, household and other community level variables. Residence in a community with higher participation of women in household decisions increased the odds of using LARP methods by ***37***% (OR, 1.37; 95% CI, 1.03–1.83) compared to residence in communities where women’s participation in household decisions is low. Similarly living in clusters or communities with higher knowledge of LARP methods significantly increased women’s odds of using LARP methods. But, living in communities with higher mean desired number of children and lower median age at marriage did not show significant association with the uptake of LARP methods after adjusting for the effects of individual and household covariates. Corresponding to the regional variation in the uptake of contraception in Ethiopia, the regression analysis also showed that the odds of using LARP methods is significantly lower (OR, 0.45; 95% CI, 0.31–0.64) in the regions that are named emerging and characterized by pastoral nomadic way of life compared to the regions that are dominantly rural and agrarian areas. The uptake of LARP methods did not vary between the urban and mixed farming rural regions however.

## Discussion

This study aimed to examine the contextual factors that influence the use of long acting reversible and permanent (LARPs) contraception among married women in Ethiopia. Expanding access to LARP contraception offers promising opportunities for addressing the high and growing unmet need for modern contraceptives in countries such as Ethiopia where nearly a quarter of married women have an unmet need for family planning. Despite the rapidly increasing use of modern contraceptive methods over the last two decades, contraceptive methods mix is largely dominated by one method, injectables, which accounted for 64% of the contraceptive method mix in 2016. The rapid increase in contraceptive use and the uptake of injectable in particular was related to the expansion of the Health Extension Program (HEP) that was launched in 2003. Access to health services in general and family planning in particular was limited in the largely rural parts of the country until the government embarked on the health extension program [37]. Cognizant of the shortage of qualified medical personnel, Ethiopia pursued task shifting / task sharing strategy with the training and deployment of HEWs and mid-level health workers since 2009 with training on implanon insertion. This has led to a significant increase in the uptake of contraceptive implants since 2010. Further, the Ministry of Health since 2016 is training HEWs, specifically Level 4 Health Extension Workers (L4HEWs) to provide IUDs and implants in the remote and rural areas of the country[35]. Nonetheless, more efforts should be made to achieve the target set by revised reproductive health strategy that envisions to increase the share of long acting reversible and permanent methods to 50% by 2020 [33].

Although contraceptive use improved over the last two decades, equity and quality concerns still remain major challenges to family planning program in Ethiopia. The disparity between the richest and poorest quintiles and between the regional states in the use of modern contraception and more importantly in choice of long acting reversible and permanent contraception is remarkable. In 2016, modern contraceptive use varied from 47% among the richest quintile to 20% among the poorest. Regionally, modern contraceptive use ranged from 56% in Addis Ababa to less than 2% in the Somali Region [28]. Similar variation is observed in the uptake of long acting reversible and permanent contraception by education and other socio-economic factors. Evidence from the recent DHS also indicates that fewer women who use modern contraception were informed about the method’s side effects, what to do if they experience side effects and other available contraceptive methods [28]. Moreover, contraceptive discontinuation is common with over a third of episodes discontinued within 12 months[28]. The country’s Health Sector Transformation plan of 2015–2020 articulates the gaps in equity and quality of health services and has set goals and strategies of addressing equity and quality issues [38].

Other major challenges to the uptake of LARP methods include low community awareness, myths and misconceptions related to the methods [19, 21, 39]. Although knowledge of any method of contraception is nearly universal in Ethiopia, knowledge of long acting reversible and permanent methods is relatively low. In 2016, only 46% and 36% of married women reported knowledge of IUD and female sterilization respectively. Knowledge of implants is relatively higher at 76% [8].

Regression analysis identified several contextual individual, household and community level factors that influence women’s choice of long acting reversible and permanent contraception. Household wealth status, women’s employment and fertility preferences were among the household and individual level variables that showed significant association with the choice of LARP methods. The disparity in access to contraception (including LARP methods) with wealth status is remarkable suggesting that Ethiopia’s family planning program has to prioritize targeted intervention that benefits the poor and marginalized sections of the population. The lack of association with education was surprising given the well documented influence of education on contraceptive behavior. The association with employment shows the benefits of employment in modern sector in terms of increasing access to information and services. Previous studies have also documented similar disparity in contraceptive use and use of long acting reversible methods by women’s occupation in Ethiopia and other parts of the world [25, 40].

Moreover, women’s fertility preference was a strong predictor for their choice of LARP methods with women desiring to space or limit child bearing showing higher odds of using LARP methods. This is expected given that the demand for contraception is higher among these categories of women. The association with age showed that adolescents and older women 40–49 years were less likely to utilize LARP methods compared to women in their 20’s. A 2013 study by Asnake and colleagues showed that the provision of Implants at the community level through task shifting to HEWs was more effective in reaching women in the 20’s who have fewer children than older women [39].

The multi-level regression analysis also identified several key contextual community level factors that influence the use of LARP methods. Exposure to family planning information through media, knowledge of LARP methods, perceived distance from health facilities, community level women’s empowerment and region were important predictors of LARP methods. The study showed that living in communities with access to family planning information and services is important for the uptake of LARP methods. Access to information about modern contraception is important due to the fact that modern contraception uptake is hindered by misconceptions, misinformation, and misinterpreted side effects [33, 34]. The use of mass media can be an important strategy in the efforts being made to increase awareness and use of LARP methods in Ethiopia [41]. However, contact of women with family planning providers (being visited by health workers at home) was not associated with choice of LARP methods in this study probably because the community health workers provide more information about the importance of family planning and short-acting methods than LARP methods. Living in clusters or communities with higher knowledge of LARP methods appears to significantly increase women’s odds of using LARP methods.

The study also showed that living in clusters with higher women’s participation in household decision-making is important for the uptake of LARP methods. A number of studies from Ethiopia and elsewhere showed that women’s empowerment is associated with their ability to seek health services [25, 42, 43]. A recent analysis of DHS data from Rwanda and Nepal by Wang and colleagues [25] also found that the extent to which women in the community participate in decisions around family planning appeared to be positively associated with women’s contraceptive use. Improving women’s autonomy enables women to make decisions about contraceptive use. The association of LARP use with development region reflected the huge disparity in the uptake of modern contraception in Ethiopia. Regions that are dominantly rural and inhabited by pastoralist populations do not only lack access to health services but are also mobile in their way of life to make use of existing health services available to them. Our findings indicate that more should be done to reach women in these rural and hard to reach pastoralist communities.

This study has some limitations. First, the study used DHS data which is cross-sectional in nature and hence the data may potentially suffer from recall bias. This design also prohibits assessment of potentially causal relation of variables. Moreover, the use of proxy variables of access to services and other cluster level variables have a limitation in that these may not adequately represent the reality on the ground. However, the use of advanced statistical techniques such as multivariate, multilevel analysis enables us to estimate robust standard errors while adjusting for the effects of individual and household level factors.

Despite the aforementioned limitations, this study adds to the body of evidence on the importance of examining community influences on contraceptive behavior to understand how contraceptive behaviors are influenced by factors beyond the individual and household-level. The findings of this study indicate that the demand for long-acting reversible and permanent contraception services in Ethiopia is influenced not only by women’s individual and household socioeconomic characteristics but also by the community’s level of women’s empowerment, socio-economic development, as well as its access and exposure to family planning information and services. Thus, improving knowledge of long-acting acting and reversible methods, improving women’s decision making autonomy and upgrading the capacity and skills of health extension workers, nurses and midwives on the provision of LARP methods and rights-based approach to the full spectrum of family planning methods with emphasis on counseling on long- acting methods are important to improve the uptake of LARP.

## Supporting information

S1 Table Bivariate multilevel logistic regression showing the association between various individual, household and cluster level variables with choice of LARP methods, Ethiopia 2016. (DOCX)

## Abbreviations

CPR: Contraceptive Prevalence Rate
CSA: Central Statistical Agency, Ethiopia
CYP: Couple years of Protection
DHS: Demographic and Health Surveys
FGAE: Family Guidance Association of Ethiopia
HEP: Health Extension Program
HEW: Health Extension Workers
HSDP: Health Sector Development Programme
ICC: Intra Cluster coefficients
IUD: Intrauterine Device
L4HEWs: Level 4 Health Extension Workers
LARP: Long-acting reversible and permanent
LMICs: Low and Middle Income Countries
MOH: Ministry of Health, Ethiopia
SSA: sub-Saharan Africa
TFR: Total Fertility Rate
