# Supplementary material for: Contextual influences on the choice of long-acting reversible and permanent contraception in Ethiopia: A multilevel analysis
Source: PLoS One. 2019 Jan 16;14(1):e0209602. doi: 10.1371/journal.pone.0209602 (PMC6334991; doi:10.1371/journal.pone.0209602)
Supplement: S1 Table — (DOCX) [file pone.0209602.s001.docx]

Additional documents

Table 4: Bivariate multilevel logistic regression showing the association between various individual, household and cluster level variables with choice of LARP methods, Ethiopia 2016

| **Variables** | Unadjusted  OR (95% CI) |
| --- | --- |
| **Individual and household variables**  **Age** (ref=20-29 years)  15-19 years  30-39 years  40-49 years  **Education** ( ref= no education)  Primary education  Secondary and above  **Residence** (RC=urban)  Rural  **Wealth** ( ref= poorest)  poorer  middle  richer  richest  **Employment** (ref= not working)  working but not paid in cash  working and paid in cash  **Parity** (ref==up to1 child)  2-3  4-5  6+  Fertility preference (ref= Want soon)  Wants later  Wants no more  Undecided/unsure  Participation in household decision making (ref= no)  Yes | 0.41(0.25-0.66)**  1.02(0.82-1.22)  0.72(0.53-0.96)**  1.23(0.99-1.53)  1.72(1.33-2.22)**  0.31 (0.23-0.41)**  2.65(1.85-3.80)*  3.53(2.47-5.05)**  3.61(2.49-5.20)**  6.42(4.61-8.94)**  1.16(0.92-1.47)  1.82(1.47-2.46)**  1.27 (1.02-1.57)*  0.98(0.76-1.26)  0.84(0.63-1.11)  2.25(1.74-2.90)**  2.42(1.85-3.17)**  2.79(1.81-4.28)**  1.40 (1.14-1.73)** |
| **Access to family planning information and services**    Exposure to family planning  information (RC =no)  Yes  Visited by FP worker in the last 12  months (RC=no)  Yes  Distance to health facility (Rc= not a  big problem)  Distance is a big problem  Knowledge of LARP methods (Rc=low)  High knowledge | 1.96 (1.61-2.38)**  1.22 (0.93-1.36)  1.63 (1.34-1.99)**  11.64 (8.11-16.71)** |
| **Community**/***Cluster variables***  Development region  (RC= rural agrarian)  Emerging/nomadic regions  Urban  Community poverty  (RC=high )  Low poverty  Community level women’s education  (RC= Low)  High  Women’s empowerment (Rc=low)  High empowerment  Community level fertility norms  (Rc= high desired # of children)  Lower desired # of children  Median at marriage (RC<17.0 years)  Median age above 17 years | 0.04(0.02-0.10)**  2.09 (1.57-2.77)**  3.50(2.65-4.63)**  3.08(2.34-4.05)**  3.72(2.76-5.04)**  0.41(0.31-0.54)**  1.74(1.32-2.30)** |
